# Brachytelephalangic chondrodysplasia punctata caused by new small hemizygous deletion in a boy presenting with hearing loss

**DOI:** 10.1186/s13039-015-0187-7

**Published:** 2015-10-31

**Authors:** Irena Vrečar, Gorazd Rudolf, Borut Peterlin, Luca Lovrecic

**Affiliations:** Clinical Institute of Medical Genetics, University Medical Centre Ljubljana, Slajmerjeva 3, SI-1000 Ljubljana, Slovenia

**Keywords:** X-linked chondrodysplasia punctata, CDPX1, ARSE, Syndromic hearing loss

## Abstract

X-linked recessive type chondrodysplasia punctata (CDPX1) is a congenital disorder of cartilage and bone development with typical findings of stippled epyphises, nasomaxillary hypoplasia and short distal phalanges in a male patient. Disease is caused due to the loss of arylsulfatase E activity and only 55 patients with genetically confirmed disease have been reported so far. In 60–75 % of all patients the mutation in *ARSE* gene is detected by sequence analysis and in further 25 % of patients Xp deletions or rearrangements are causative and may be identified by classical chromosome studies. We report on a male patient refered to clinical geneticist for congenital hearing loss and mild dysplastic signs, both phenotypic features being relatively unspecific and non suggestive of CDPX1 in first instance. Array comparative genomic hybridisation showed approximatelly 3 kb big deletion, spaning intron and exon 7 of arylsulfatase E gene located in Xp22.33. This explained the cause of hearing loss, being present in 26–89 % od CDPX1 patients, as well as additional non prominent skeletal characteristics described by geneticist in our patient - mild midface hypoplasia and mild brachytelephalangy. Reported case introduces different presenting clinical phenotype for CDPX1, emphasizing different expressivity in this disorder.

## Background

Chondrodysplasia punctata (CDP) is a heterogeneous group of bone disorders, clinically and genetically diverse. Typical characteristic is punctiform calcification of the bones. With different combination of additional features, there are now 5 forms with known genetic cause. No classification consensus has been established. There are 3 autosomal recessive rhizomelic forms (RCDP1 OMIM#302960, RCDP2 OMIM#222765 and RCDP3 OMIM#600121), one X-linked dominant form (CDPX2 OMIM#215100) and here reported and reviewed X-linked recessive CDPX1 (OMIM#302950), consequence of mutations in *ARSE* gene (OMIM*300180) at Xp22.33. Due to the characteristic short distal phalanges, it is reffered to as brachytelephalangic chondrodysplasia punctata (BCP). Typical findings in this group of skeletal dysplasia are stippled epyphises (chondrodysplasia punctata), nasomaxillary hypoplasia (Binder phenotype) and short distal phalanges (brachytelephalangy). Interestingly, both acquired and genetic causes have been described, presenting with similar phenotype [1, 2]. Metabolic abnormalities include disorders in peroxisomal metabolism, lysosomal storage disorders, disturbed cholesterol biosynthesis and disruption of vitamin K metabolism. Gene ARSE codes for arylsulfatase-E, vitamin K dependent enzyme, and its activity is inhibited in the presence of warfarin, an anticoagulant drug that decreases amounts of active vitamin K. Therefore, warfarin exposure and vitamin K deficiency present logical phenocopies of CDPX1 and there are case reports described confirming this [2, 3]. In addition, it was known already in late 1970s that warfarin treatment during pregnancy can cause nasal hypoplasia, stippled epiphyses and vertebral defects in fetuses [4].

The *ARSE* gene is located at Xp22.33, within a cluster of contiguous arylsulfatase genes, that share high sequence homology, escape X inactivation, and have pseudogenes on the Y chromosome [5]. ARSE is localized to the Golgi membranes and its transcript has been identified in multiple tissues [6]. Most affected males have minimal morbidity with normal intellect and life span and skeletal findings typically diminishing by adulthood. In contrast to relatively mild morbidity in most males, additional medical problems such as short stature, mixed conductive and sensorineural hearing loss, abnormal cognitive development, and possibly significant complications such as airway stenosis and cervical spine instability with compression of the cervical spine cord, have been described in some cases. Typical X-ray finding of punctate epyphises is not obligatory and frequently only present in early childhood.

By now around 140 male patients with a clinical phenotype had been reported, but genetic cause has been confirmed in less than half. In 60–75 % of CDPX1 cases, reported genetic cause is detected by sequence analysis of *ARSE* gene, in further 25 % of patients Xp deletions or rearrangements are identified on classical chromosome studies [6]. Smaller deletions can be evaluated by array CGH. In the group of patients with deletions, only one case of single exon deletion has been reported, deletion of exon 10 in a severly affected patient [7]. There is a case of deletion of exons 1 and 2 with severe clinical presentation as well [3]. Appart from that, only large deletions, including the whole ARSE gene had been reported [8]. All the mothers of genetically confirmed cases, that were tested, were reported to be carriers of point mutation or deletions. There is no case of confirmed de novo mutation in the literature reported so far.

Here we report a new case in the terms of clinical presentation and mutation type/size. Namely, male patient was refered to clinical geneticist for congenital hearing loss and mild dysplastic signs and array CGH showed deletion of intron and exon 7 in *ARSE* gene.

## Case presentation

### Patient

9 year old boy was reffered to Clinical Institute of Medical Genetics Ljubljana because of mixed sensorineural hearing loss and mild dysplastic signs. He was born as a first child to unrelated healthy parents after uneventful pregnancy, with normal birth parameters (birth weight at 60th percentile, birth length at 70th percentile) and APGAR score 9/9/10. Screening test for newborns showed congenital hearing loss. Brain stem acustic potential evaluation identified mixed sensorineural hearing loss at age 13 days. General examination revealed nasomaxillary hypoplasia and mild hypotonia, which persisted until one year of age. Further medical exams including brain ultrasound, abdominal ultrasound, CT of temporal bones and eye examination revealed no further abnormalities. All developmental milestones where achieved as expected. Family history was uneventful for genetic diseases, skeletal abnormalities, congenital anomalies and hearing loss. At the age of 9 years, clinical geneticist described high forehead, midfacial hypoplasia with mild hypertelorism, hypoplastic nose, depressed nasal bridge and long philtrum.

### Genetic testing

**Fig. 1:**
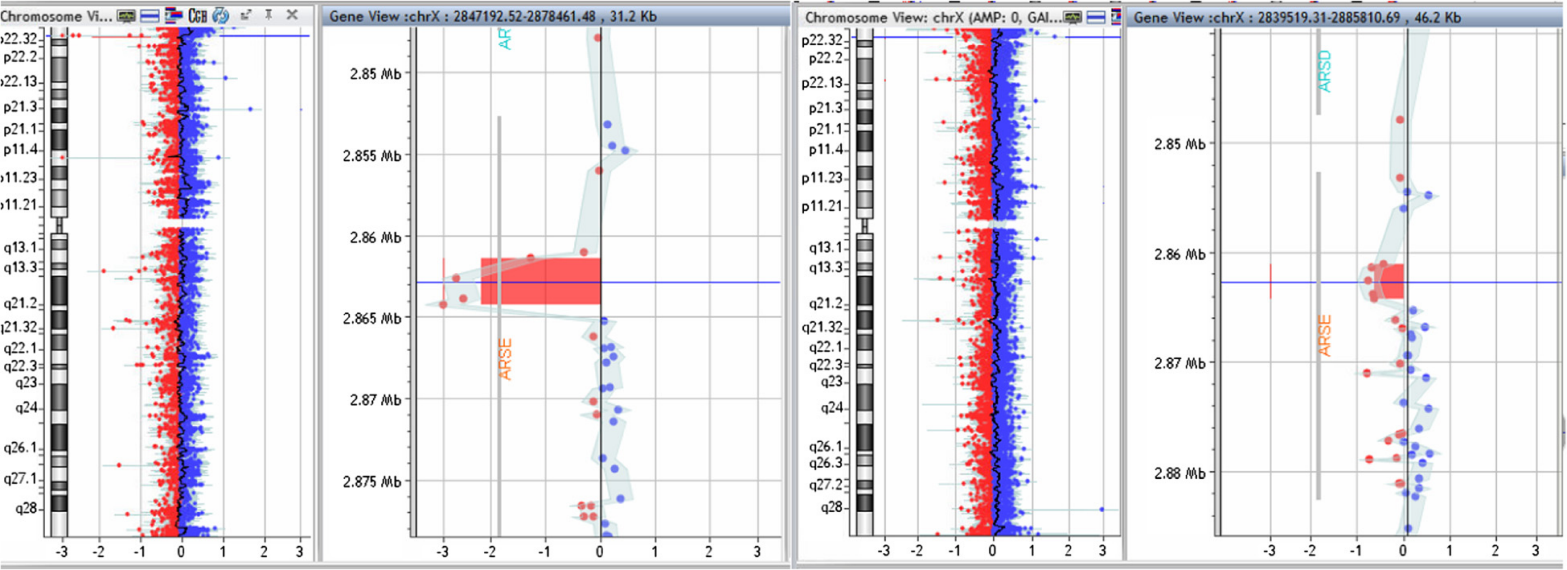
Array CGH results in reported case (left panel) and his mother (right panel). Hemizygous deletion in the boy shows higher relative number values (between −2 and −3) compared to heterozygous deletion in the mother (around −0.8 and −1)

Chromosome analysis in 2005 showed normal male karyotype 46, XY. Molecular genetic analysis for hearing loss in 2008, ordered by otorhinolaryngologist, monitoring the boy's hearing, did not identify any mutations in *GJB2* and *GJB6* genes, which excluded the most common cause of genetic nonsyndromic hearing loss. Namely, mutations in mentioned genes are reported to be found in 33,0 % of children with hearing loss in Slovenia [9]. After seeing the patient when he was 9 years old, array CGH was performed and showed minimum 2886 bp large deletion, spaning intron and exon 7 of *ARSE* gene (arr[hg19] Xp22.33(2,861,119×1,2,861,384-2,864,270×0,2,865,276×1)). Proximal breakpoint and next available microarray probe was only 265 bp away, distal next available probe was 1006 bp away. Mother was deletion carrier, as well (Fig. 1)

.

### Overview of reported genetic variants

There are 55 patients with CDPX1 reported so far [8]. Seven patients have deletion of the full ARSE gene, one patient has exon 1–2 deletion, 2 patients (brothers) have exon 7–10 deletions and one has only exon 10 deletion. Together with here reported case, 12/56 (21 %) cases have partial or whole gene deletions. There is one reported case with triplet deletion in exon 3 and two cases with duplication of single nucleotide. All others have point mutations. Vast majority of reported mutations are private family mutations (Table 1). Although reported mutations are distributed throughout the gene, except for exon 1 and 2 where there is no reported point mutations, it seems that mutations in exon 5 and exon 11 are more frequently reported. When looking at reported cases with point mutation/single nucleotide changes, there are 7/43 (16 %) and 14/43 (33 %) reported cases with mutation in exon 5 and exon 11, respectively.

**Table 1:** Reported mutations in ARSE gene

Exon	Mutation	Number of patients	Inherited
00_02i	Exons 1–2 deletion	1	+
00_11	Whole gene deletion	7	+
3	c.36G > C	1	+
3	c.119 T > G	2	+
3	c.126_128delTCT	1	NA
3	c.169G > A	1	NA
*02i_03i*	*Exon 3 deletion*	*1*	*+*
4	c.217G > A	1	+
4	c.239 T > A	1	+
4	c.268A > G	1	NA
5	c.314dupT	1	NA
5	c.332G > C	1	+
5	c.349G > A	1	NA
5	c.345G > A	1	NA
5	c.410G > C	3	+
5	c.410G > T	1	NA
6	c.445G > T	1	NA
6	c.733G > C	1	NA
6	c.767dupT	1	+
06i_10i	Exons 7–10 deletion	2	+
7	c.916A > G	1	NA
7	c.949G > A	2	+
8	c.1063G > A	1	NA
9	c.1130G > A	2	+
9	c.1171G > A	1	+
9	c.1226C > T	1	+
09i_10i	Exon 10 deletion	1	NA
10	c.1300G > A	2	+
10	c.1387G > A	1	NA
11	c.1442C > T	4	+
11	c.1475G > A	1	NA
11	c.1618C > T	1	NA
11	c.1732C > T	2	+
11	c.1743G > A	6	+

## Discussion and conclusion

Typical combination of signs of CDPX1 is stippled epyphises, nasomaxillary hypoplasia and short distal phalanges in a male patient. This was not so obvious in our patient, who was refered to us because of congenital hearing loss and mild dysplastic signs (those being in concordance with typical signs of CDPX1) and was found to have a small intragenic ARSE deletion by array CGH. At the time of examination, we would not expect the signs of chondrodysplasia punctata, as it can be observed already in the second trimester of pregnancy, but tends to improve and even disappear by age 2–3 years.

The disease is relatively rarely reported - there are only 56 described patients, including our case - and phenotypic spectrum is broad. One might speculate that it is different, according to the type, location and size of genetic defect. About 1/5 of cases have partial or whole gene deletions detectable with array-CGH and previously reported larger deletions with classical cytogenetics. The remainder of genetically confirmed cases have point mutations or single nucleotide insertions and therefore need sequence analysis to confirm the genetic cause. About 1/3 of reported point mutation/single nucleotide changes are located in exon11. There are two possible explanations - firstly, exon might be more prone to mutations due to so far unknown reasons or, more likely, mutations in exon11 present with more specific and severe clinical symptoms and therefore bring proportionally more affected males to medical attention and further investigations. To support this, there are six reported cases with severe symptoms and confirmed single nucleotide ARSE mutation so far [7, 10], all mutation being in exon 11. Three born patients had significant respiratory distress requiring assisted ventilation due to cervical spinal canal stenosis and related spinal cord compression. Three cases were aborted in late second trimester when severe spinal canal stenosis was seen. There are 2 additional clinical reports of newborn males, both with cervical spinal canal stenosis and typical radiographic chondrodysplasia punctata, flat nasal bridge and brachytelephalangy, but no molecular genetic testing has been performed [11].

On the other side of phenotypic spectrum, there are mild cases. Typical radiographic signs (punctate epyphises) are frequently only present in early childhood and after that only minor signs might be present and therefore go unrecognised. Some cases were described with only shortened distal phalanges and mild midfacial hypoplasia [1]. Delayed motor and cognitive development are reporeted in 19–33 % and 16–20 % of cases, respectively [3, 6]. Also, fertility is normal. For both reasons, family mutations might spread to next generations, not only through healthy female mutation carriers, but through affected (undiagnosed) males, as well. It was reported in a family with mild phenotypic presentation, that maternal uncle with no major health issues, was a mutation carrier as well [12]. Unfortunatelly, other family members were not available for testing in our case.

In addition to genetic cause, important maternal etiological factors have been linked to the phenotype (warfarine exposure, vitamin K deficiency, maternal autoimmune disease), but still around 40 % of male patients with brachytelephalangic chondrodysplasia punctata do not have detectable *ARSE* mutations or known maternal etiological factors [3, 6]. Further understanding of mechanism behind ARSE function might prove useful in identifying other potential candidates in the same disturbed pathway that, when defective, would lead to the similar phenotype of brachytelephalangic chondrodysplasia punctata. As already mentioned, these may involve maternal-fetal vitamin K deficiency, with warfarin embryopathy being known for decades. Already in late 1970s it was reported that fetuses of women treated with warfarin during pregnancy, are at increased risk for nasal hypoplasia, stippled epiphyses and vertebral defects [4]. Maternal systemic lupus erythematosus has been reported to be a risk factor for the same phenotype in fetus, as well [13]. Not all fetuses exposed to warfarin or born to mothers with SLE develop CDP typical signs, and further knowledge of mechanisms behind this phenotype is needed to help elucidate all the causes behind it.

To conclude, we present a case of X-linked recessive chondrodysplasia punctata (CDPX1), interesting for two reasons. Firstly, this is the first case, diagnosed with array CGH and found to have a deletion of intron7 and exon7 in ARSE gene. Secondly, this is also the first case to present with congenital sensorineural hearing loss and mild facial dysmorphisms at the age of 9 as major clinical signs. With this case we add to clinical and genetic spectrum of CDPX1.

## Consent

Informed consent was obtained from the patient’s mother for publication of this Case report. She did not agree with publication of images.
